# Therapeutic perspectives for adult soft tissue sarcoma—updates from the 2022 ASCO annual meeting

**DOI:** 10.20892/j.issn.2095-3941.2022.0394

**Published:** 2022-11-01

**Authors:** Jilong Yang, Yu Xu, Yong Chen, Tao Li, Xiaowei Zhang, Tu Hu, Ruwei Xing, Yun Yang

**Affiliations:** 1 Department of Bone and Soft Tissue Tumor, Tianjin Medical University Cancer Institute & Hospital, National Clinical Research Center for Cancer, Key Laboratory of Cancer Prevention and Therapy, Tianjin, Tianjin’s Clinical Research Center for Cancer, Key Laboratory of Molecular Cancer Epidemiology, Tianjin 300060, China; 2 Department of Musculoskeletal Surgery, Fudan University Shanghai Cancer Center, Fudan University, Shanghai 200032, China; 3 Department of Bone and Soft tissue Surgery, Zhejiang Cancer Hospital, Hangzhou 310022, China; 4 Department of Gastrointestinal Medical Oncology, Fudan University Shanghai Cancer Center and Department of Oncology, Shanghai Medical College, Fudan University, Shanghai 200032, China

At the recent 2022 American Society of Clinical Oncology (ASCO) annual meeting, the latest progress was presented in clinical trials of various therapeutic modalities for adult soft tissue sarcoma (STS), including chemotherapy, targeted therapy, anti-immune checkpoint immunotherapy, and multiple combination treatments (**Table 1**). Generally, the development of clinical treatments for STS is relatively slow, owing to the complex pathological subtypes of sarcoma and their heterogeneous biological behaviors. Here, we briefly summarize updates from this year’s ASCO meeting and discuss the future therapeutic perspectives for unspecific STS.

**Table 1:** Key reports on adult soft tissue sarcomas at the 2022 ASCO meeting

Abstract No.	Regimen	Sarcoma subtype	Treatment line	Patient number	Result
11508	Epirubicine + ifosfamide(EI), trabectedin(T)	MLPS	Neoadjuvant	101	AI *vs*. T: 5 yr-DFS 86% *vs.* 73%, 5 yr-OS 88% *vs*. 90%
11575	Trabectedin	Retroperitoneal LPS or LMS	Post-line	91	LMS: ORR 24%, TTP 6 mos; LPS ORR 12%, TTP 6 mos
11507	Unesbulin (PTC 596), dacarbazine	LMS	Post-line	33	ORR 18.2%, RP2D cohort, 5/6 PR
11555	Regorafenib	LMS, SS, and other STS	Post chemotherapy or pazopanib	175	PFS 2.1 mos, OS 14.4 mos
11557	Surufatinib	LMS, SS, UPS, and other STS	Post-line	32	6 SD, PFS 2.56 mos, 4 mos-PFS 17.5%
11506	Lenvatinib, eribulin	LPS, LMS	Not specific	30	6 PR, 21 SD, ORR 20%, PFS 8.56 mos, OS 27 mos, 1 yr-OS 89%
3004	BI 907828 (MDM2-p53 antagonist)	LPS (DDLPS, WDLPS) and other solid tumors with TP53 wild-type	Post-line	96 (49 LPS)	DDLPS *n* = 34, 4 PR, ORR 12.5%, DCR 87.5%, PFS 8 mos; WDLPS *n* = 15, 4 PR, ORR 26.7%, DCR 100%, PFS NR
11509	Olaparib, temozolomide	Uterine LMS	Post-line	22	6 PR, 9 SD, ORR 27%, DCR 68%, DoR 12 mos, PFS 6.9 mos, OS NR
11503	Prexasertib (CHK1/2 inhibitor), irinotecan	DSRCT and RMS	Post-line	21	DSRCT cohort *n* = 19, ORR 32%, PFS 24 wks; RMS cohort *n* = 2 2 SD
LBA11501	Nivolumab, nivolumab + ipilimumab	Retroperitoneal DDLPS, extremity/truncal UPS (with neoadjuvant radiation)	Neoadjuvant	DDLPS 17; UPS 10	DDLPS cohort: 8.8% hyalinization, RFS 17.5 mos, 2-yr RFS 35%, 2-yr OS 82% UPS cohort: 89% hyalinization, RFS NR, 2 yr-RFS 70%, 2 yr-OS 100%
11573	Ipilimumab, nivolumab, trabectedin	LMS, LPS, UPS, and others	First-line	101	ORR 26.6%, DCR 87.3%, PFS 6.7 mos, OS 24.6 mos
11516	Gemcitabine, docetaxel, retifanlimab (R)	STS	First-line	13	R 270 mg cohort: ORR 17%, DCR 100%, 24 wks-PFS 60%, PFS 24.3 wks; R 375 mg cohort: ORR 50%, DCR 83%, 24 wks-PFS 44%, PFS 22.3 wks
11500	TCR-T lete-cel; GSK3377794 NY-ESO-1 HLA-A*02:01, *02:05, *02:06	MRCLS	Post-line	Cohort 1 *n* = 10; Cohort 2 *n* = 10	Cohort 1 (reduced-dose lymphodepletion) *n* = 10, ORR 20%, DoR 5.31 mos, PFS 5.36 mos; cohort 2 (standard-dose lymphodepletion) *n* = 10, ORR 40%, DoR 7.47 mos, PFS 8.74 mos
11502	TCR-T TAEST16001; NY-ESO-1 HLA-A*02:01	SS (major) and others	Post-line	12	5 PR, 5 SD ORR 41.7%, DoR 14.1 mos, PFS 7.2 mos
11562	TCR-T Afami-cel; SPEAR T cell MAGE-A4 HLA-A*02	SS, MRCLS	Post-line	69	ORR 36.2%, DoR 52 wks. SS: responders PFS 58.3 wks, 24 wks-PFS 80%; non-responders PFS 11 wks, 24 wks-PFS 20%

ORR, overall response rate; DoR, duration of response; PFS, progression-free survival; OS, overall survival; MRCLS, myxoid/round cell liposarcoma; SS, synovial sarcoma; LMS, leiomyosarcoma; DDLPS, dedifferentiated liposarcoma; UPS, undifferentiated pleomorphic sarcoma; STS, soft tissue sarcoma; IPI, ipilimumab; LPS, liposarcoma; WDLPS, well-differentiated liposarcoma; RP2D, recommended phase II dose; DSRCT, desmoplastic small round cell tumor; RMS, rhabdomyosarcoma; MLPS, myxoid liposarcoma; TTP, time to progression.

## Chemotherapy

Through a variety of clinical trials and explorative research, traditional cytotoxic chemotherapy with anthracyclines (mainly doxorubicin or epirubicin) and ifosfamide (AI) remains the first-line regimen for most of adults with STS, regardless of histopathology type. Other drugs, including gemcitabine, docetaxel, dacarbazine, trabectedin, and eribulin, are selected as secondary or post-line setting treatments.

The STS 1001 neoadjuvant study, conducted by Italian, Spanish, French, and Polish sarcoma research groups, and published in 2020, has indicated that 3 pre-operative cycles of AI chemotherapy resulted in better survival outcomes than histotype-tailored settings in 5 major high-graded sarcoma subtypes. However, in the high-grade myxoid liposarcoma (MLPS) subgroup, neoadjuvant trabectedin had similar disease-free survival (DFS, HR 1.03) and overall survival (OS, HR 1.05) to those of an AI regimen^1^. In this year’s meeting, an update was provided on an expansion study of high-grade MLPS with a non-inferiority design. With a median follow-up of 66 months, AI and trabectedin had comparable DFS (86% *vs*. 73%, *P* = 0.26) and OS (88% *vs.* 90%, *P* = 0.90) outcomes^2^. Meanwhile, trabectedin showed a better safety profile with much less hematological toxicity and slightly increased liver toxicity, which was primarily grade 1 or 2^2^. Moreover, neoadjuvant chemotherapy conferred an OS benefit for patients with MLPS, compared with the survival threshold predicted by the nomogram in the validated Sarculator tool^3^.

Beyond trabectedin, other new biological compounds including anti-microtubule agents such as eribulin and unesbulin, have been reported to treat certain STS subtypes, mainly leiomyosarcoma (LMS) and liposarcoma (LPS)^4,5^. A phase II study of trabectedin on retroperitoneal LPS and LMS, and a phase Ib study introducing a new microtubular inhibitor, unesbulin, combined with dacarbazine for LMS, were also presented at this year’s ASCO meeting, thus suggesting promising anti-tumor roles of these treatments^6,7^.

In 2022 ASCO meeting, Zhou et al., at Zhongshan Hospital, Fudan University, China, reported the efficacy of eribulin among Chinese STS patients in real-world practice. The overall response rate (ORR) values for 10 patients receiving eribulin monotherapy and 30 patients receiving combination therapy were 50% and 20%, respectively^8^. The PFS was approximately 4 months in both groups. Among sarcoma subtypes, for L-type sarcoma (LMS/LPS) and non L-type sarcoma, the ORR was 30.3% and 14.3%, respectively, and the PFS was 4.5 months and 2.7 months, respectively^8^. The best clinical benefit was observed in MLPS, with an ORR of 54.5% (6/11) and disease control rate (DCR) of 72.7% (8/11)^8^.

## Targeted therapy

Multi-target small molecular tyrosine kinase inhibitors (TKI; mainly targeting VEGFR, FGFR, KIT, etc.) have been developed and approved for refractory STS in multiple regions worldwide; examples include pazopanib, regorafenib, and anlotinib. However, in 2 clinical trials presented this year, regorafenib and surufatinib both showed a very short PFS of approximately 2 months^9,10^. The specific anti-VEGFR2 TKI drug apatinib also showed encouraging efficacy in post-line treatment for metastatic STS. In our previous report on apatinib in treatment of metastatic STS after failure of chemotherapy, the best ORR, evaluated at 12 weeks, was 26.32% (10/38), and an mPFS of 7.87 m was achieved^11^.

In combination treatments with chemotherapy, an update was provided on a phase Ib/II study of lenvatinib and eribulin in advanced LPS and LMS by researchers from Taiwan, China. The combination therapy achieved a 20% ORR and a median PFS of 8.6 months^12^. Interestingly, patients who received this therapy as first-line treatment appeared to achieve no further benefit, but had poorer response rates and disease control rates, thus suggesting that this treatment cannot yet challenge front-line treatment with AI and targeted therapy^12^.

Recently, progress has been made in advancing other directions in targeted therapy. Beyond previously reported epigenetic drugs such as EZH2 inhibitors and HDAC inhibitors, novel agents targeting genes in the MDM2-TP53 pathway and DNA damage repair pathway emerged in this year’s ASCO meeting. BI907828, a highly potent oral MDM2-p53 antagonist, has been investigated in a phase I study in solid tumors, including advanced or metastatic LPS^13^. This modality has shown promising efficacy in both de-differentiated and well-differentiated LPS, with an ORR of 12.5% and 26.7%, respectively, and a DCR of 87.5% and 100%, respectively^13^. In last year’s ASCO meeting, a phase II study on olaparib combined with temozolomide in advanced uterine LMS was reported. This year, an update on the clinical benefits, as well as correlative results on biomarker exploration, was presented. With longer follow-up, the ORR increased slightly, to 27% overall, and the DCR was 68%, with a median PFS of 6.9 months and a duration of response (DoR) of 12 months. Four patients remained on treatment for more than 2 years^14^. A translational study indicated that genomic alterations in the homologous recombination pathway, detected by whole exome sequencing, and a lack of RAD51 foci formation in baseline tumor samples had positive predictive value in determining the clinical benefit of the combination treatment^14^. Furthermore, in a phase I/II study, another novel agent targeting CHK1/2 in the DNA damage repair pathway has been assessed in combination with irinotecan for refractory desmoplastic small round cell tumor (DSRCT) and rhabdomyosarcoma (RMS). In the DSRCT cohort, the ORR reached 32%, and all 2 patients with RMS achieved stable disease as the best response^15^.

## Anti-immune checkpoint immunotherapy

Responses to immune checkpoint blockade therapies vary widely among heterogeneous subgroups of STS. Generally, sarcoma is treated as an immune “cold” tumor, because its cold tumor microenvironment usually does not respond to immunotherapy. However, previous studies have demonstrated that a high density of B cells and the presence of tertiary lymphoid structures (TLS) in tumors might be associated with better response rates to immunotherapy^16^. In the PEMBROSARC study, after recruitment of patients with TLS detected by immunohistochemistry on baseline tumor samples, the ORR for anti-PD1 monotherapy (pembrolizumab) increased to 30%, and the PFS was extended to 4.1 months^17^.

Updates on survival and a biomarker study of neoadjuvant nivolumab monotherapy or combination therapy with ipilimumab on resectable retroperitoneal LPS and extremity/truncal UPS were presented at this year’s ASCO meeting. Patients with UPS received radiotherapy before the surgery. Intratumoral B-cell infiltration, TLS formation, and increased diversity and clonality of the intratumoral BCR repertoire at baseline were favorable prognostic factors for immunotherapy^18^. However, in contrast to the minimal pathologic response in the retroperitoneal LPS group receiving only immunotherapy, nearly 90% pathological hyalinization was observed in the UPS group after immunotherapy and radiation, thus suggesting that radiologic therapy might contribute to pathologic response in neoadjuvant immunotherapy treatments for UPS. The long term benefits of this neoadjuvant immunotherapeutic treatments remain to be confirmed in randomized controlled trials.

Efforts have been made to enhance immunotherapy by combing chemotherapy for STS in the past few years. Evidence of a synergistic interaction between these treatment types remains lacking. Survival updates for phase 2 trials using nivolumab, ipilimumab, and Trabectedin for previously untreated advanced STS were reported in this year’s ASCO meeting, with an ORR of 26.6%^19^. Another combination of PD-1 inhibitor retifanlimab with chemotherapy with gemcitabine and docetaxel as first-line therapy in a phase I/II study was associated with a maximum ORR of 50% and a DCR of 100%^20^. However, the median PFS associated with both combinations above did not show a significant improvement and was limited to only 4–6 months.

A combination of VEGFR-TKI and anti-PD1 immunotherapy as a second-line treatment has also been widely explored in clinical trials as well as in real-world practice. We previously reported a retrospective study on biomarker investigation and the efficacy of anti-PD1 immunotherapy in Chinese patients with bone and soft tissue sarcoma after failure of traditional chemotherapy treatment; most patients (20/24) received camrelizumab (anti-PD1) combined with apatinib (VEGFR2-TKI)^21^. The ORR and DCR were 16.7% and 55.6%, respectively, and the PFS reached 7.59 months^21^. In translational studies of this patient cohort, we have found that conventional biomarkers, including TMB, TNB, MSI, HLA-LOH, and PD-L1 expression, and sarcoma types are not associated with clinical response. However, higher intratumoral heterogeneity, a higher percentage of immune cell infiltration, and lower stromal gene expression is detected in responders than non-responders^21^.

## Cellular therapy

Cellular therapy, particularly T cell receptor-engineered T cell (TCR-T) treatment, has made outstanding progress for adult STS, as reported in this year’s ASCO meeting. Two types of NY-ESO-1-specific TCT-T therapy developed in the US and China in HLA-A*02 patients, were introduced for advanced STS. Letetresgene atuoleucel (lete-cel; GSK3377794) had previously achieved a maximum ORR of 50% and a PFS of 14.5 months in synovial sarcoma (SS). In a pilot study on myxoid/round cell liposarcoma (MRCLS) presented this year, the ORR also reached 40%, with a PFS of 8.7 months in patients receiving standard high-dose lymphodepletion^22^. Comparatively, Chinese TAEST16001 cells, another TCR affinity enhanced specific-T-cell therapy, achieved an ORR of 41.7% and a PFS of 7.2 months in the STS group mostly comprising the SS histotype^23^. Updated data on the SPEARHEAD-1 trial of afamitresgene antoleucel (Afami-cel) in SS and MRCLS expressing MAGE-A4 indicated an ORR of 36.2% in all patients and 40.7% in the SS cohort, and a median PFS exceeding 1 year in responders^24^.

The new horizon of cellular immunotherapy has come into view for a variety of cancers. However, several issues faced in real-world clinical practice might limit the expansion and the development of cellular therapy. Among them, prolonged cytopenia due to lymphodepletion chemotherapy before T-cell infusion is a critical concern. Furthermore, previous trials have indicated that different lymphodepletion conditions might affect treatment efficacy^25^. Recently, new CAR-T cells designed with synthetic chimeric receptors with an orthogonal IL-2r extracellular domain fused with an IL-9r intracellular domain have been described, which can also be expanded in the absence of conditioning lymphodepletion^25^. This technology may support the development of more efficient, safer cellular therapies in the future.

## Future perspectives

Future perspectives in the medical treatment of soft tissue sarcomas include several dimensions. Generally, therapeutic approaches will develop in both a sarcoma subtype dependent and a sarcoma subtype agnostic manner.

For most sarcoma types, traditional cytotoxic chemotherapy, mainly with an AI regimen, remains the standard front-line treatment for advanced adult STS. Novel agents such as Trabectedin and eribulin might be applied as post-line treatments for certain histotypes. Moreover, combination treatments with these novel and conventional cytotoxic agents, or the combination of chemotherapy with other treatments including immunotherapy, anti-angiogenetic agents such as endostatin, antibodies to VEGF(R), or TKI should be further investigated in front-line settings^26^. Recently another phase III randomized trial, the LMS-04 trial, has demonstrated that doxorubicin plus trabectedin in first-line therapy, compared with doxorubicin alone, significantly increases PFS in patients with LMS^27^.

For tumor-type dependent therapy, next generation sequencing and molecular tumor board based targeted therapies should help achieve more precision treatments for STS patients. Targeting driver mutations such as BRAF, ALK, NTRK, ROS1, EZH1/2, and KIT in certain rare types of sarcomas has been reported to be associated with relatively high response rates. A recent study has validated RET as a tissue-agnostic target with sensitivity to RET inhibition by pralsetinib, with an ORR as high as 57% in RET fusion-positive solid tumors, including several subtypes of sarcoma^28^. In other sarcomas, a lack of confirmed driven mutations, or genomic aberrations in some other oncogenic regulation pathways, can be exploited for therapeutic approaches, such the D-cyclin-CDK4/6-INK4a-Rb pathway, MDM2-TP53 pathway, and BRACness in the homologous recombination DNA repair pathway. Moreover, novel compounds targeting non-mutational epigenetic alterations, such as inhibitors of IDH1, ATR, DNMT, HDACs, and BET, have also been investigated in early-phase trials in patients with sarcoma^29^.

In immunotherapy, most sarcoma subtypes, except for certain sensitive subtypes, such as alveolar soft part sarcoma, UPS, myxoid fibrosarcoma, and angiosarcoma, have low tumor immunogenicity and respond poorly to immune checkpoint blockade. In-depth investigation of the tumor associated microenvironment, particularly the immune microenvironment, has identified an “immune-high” subgroup of sarcomas characterized by elevated expression of a B cell-associated gene signature, CD8+T cell infiltration, and intratumoral TLSs^16^. Screening patients with these effective biomarkers and combining other treatments to target the tumor microenvironment, are key to improving the clinical response to immunotherapy. Moreover, individualized cellular therapy including TCR-T and CAR-T therapy might be the most effective treatment for patients with advanced and refractory disease.

Nevertheless, in real-world clinical practice, comprehensive considerations by multidisciplinary teams and molecular tumor boards (MTB) are urgently required to formulate a precise therapeutic strategy for each patient with advanced and refractory STS, particularly for patients with high-grade, multiply recurrent, or large tumors adhering to important vessels or nerves. Pre-operative treatment including systemic medication, radiation, or interventional therapy should be considered to ensure radical resection, to decrease the recurrence rate, and to improve overall outcomes. Therefore, trials of neoadjuvant therapies should be encouraged in sarcomas, to investigate the efficacy and safety of new compounds or combination treatments in relatively short periods. Moreover, biomarker explorations could be conducted through analysis of both pre- and pro-treatment tumor or liquid samples.
